# Comparison of Safety and Effectiveness Between Direct Oral Anticoagulants and Vitamin K Antagonists in Dementia Patients with Atrial Fibrillation: A Systematic Review and Meta-Analysis

**DOI:** 10.3390/jcm14165758

**Published:** 2025-08-14

**Authors:** Abdulmajeed M. Alshehri, Lama Alfehaid, Solaiman Alhawas, Abdulmajeed Alsuwaylihi, Abdulaziz Alarifi, Majed S. Al Yami

**Affiliations:** 1 Department of Pharmacy Practice, College of Pharmacy, King Saud bin Abdulaziz University for Health Sciences, Riyadh 11481, Saudi Arabia; 2 King Abdulaziz Medical City, National Guard Health Affairs, Riyadh 14611, Saudi Arabia; 3 King Abdullah International Medical Research Center, Riyadh 11481, Saudi Arabia; 4 Saudi Pharmacovigilance Group, Saudi Pharmaceutical Society, Riyadh 12372, Saudi Arabia

**Keywords:** DOAC, warfarin, dementia, atrial fibrillation

## Abstract

**Background/Objectives**: The question of whether the benefits of anticoagulation outweigh the risks of bleeding in patients with dementia and atrial fibrillation (AF) remains unresolved. This study aimed to evaluate the effectiveness of oral anticoagulation (OAC) and to compare the safety and effectiveness of direct oral anticoagulants (DOACs) with vitamin K antagonists (VKAs) within this at-risk population. **Methods**: This meta-analysis was conducted following the PRISMA guidelines and was registered in PROSPERO. Data were extracted from MEDLINE, Web of Science, and Cochrane Library. The Newcastle–Ottawa Scale was used to assess the risk of bias. The outcomes were analyzed using the Comprehensive Meta-Analysis software, with odds ratios (ORs) and 95% confidence intervals (CIs) calculated for dichotomous variables. **Results**: Eight retrospective studies were included, with sample sizes of up to 40,350 participants. The primary outcome was mortality incidence, while secondary outcomes included ischemic stroke, major bleeding, and intracranial hemorrhage (ICH). DOACs significantly reduced ICH events compared to VKAs (OR 0.38, 95% CI 0.17–0.84; I^2^ = 87.3%) but showed no significant difference in major bleeding (OR 0.48, 95% CI 0.22–1.03; I^2^ = 88%). Mortality and ischemic stroke rates were similar between the DOAC and VKA groups. OAC use reduced mortality by 29% compared to no OAC (OR 0.71, 95% CI 0.57–0.88; I^2^ = 81.3%) but increased major bleeding risk (OR 1.19, 95% CI 1.08–1.3; I^2^ = 0%). **Conclusions**: DOACs offer a safer profile regarding ICH in dementia patients with AF compared to VKAs, with no significant differences in mortality or ischemic stroke rates. This study highlights the need for careful anticoagulant selection in this vulnerable population.

## 1. Introduction

Atrial fibrillation (AF) represents the most prevalent form of arrhythmia, characterized by an irregular and frequently rapid heartbeat. In patients diagnosed with AF, disorganized electrical signals can lead to increased blood stasis within the atrium and the potential formation of blood clots, particularly within the left atrial appendage, which heightens the risk of cardioembolic stroke. Clinical guidelines recommend the use of oral anticoagulants (OAC) for most patients with AF based on their estimated annual stroke risk calculated using the CHA_2_DS_2_-VASc score. However, it is crucial that the decision to prescribe OAC is based on a thorough assessment of each patient’s individual risks and health conditions. This requires careful evaluation and ongoing monitoring [1,2].

AF is more prevalent among the elderly, particularly in individuals with multiple health conditions and varying degrees of frailty [3]. This trend presents significant clinical challenges and adversely affects overall health outcomes [4]. Additionally, AF is associated with an increased risk of cognitive decline and dementia, further complicating the management of patients dealing with both issues [5,6,7]. The treatment of these patients is particularly challenging due to concerns surrounding the administration of OAC and the increased risk of intracranial hemorrhage (ICH) [8]. Additionally, dementia often results in lower medication adherence and a higher chance of errors, potentially causing inappropriate dosing [9]. Furthermore, elderly patients with dementia are at a higher risk of major bleeding from OAC therapy due to multiple comorbidities, declining renal function, and cognitive impairments [10]. Therefore, considering the initiation or continuation of OAC for patients with dementia requires conducting a comprehensive evaluation of individual risk factors, potential clinical benefits, and the patient’s overall health status and life expectancy. Additionally, discussions should involve the preferences of both the patient and their caregivers [1].

Current research findings regarding the use of OAC therapy for AF patients with dementia have been inconsistent [11,12,13,14,15]. Moreover, it remains uncertain whether direct oral anticoagulants (DOACs) would be a better option for this patient population, considering their lower adherence requirements and potentially reduced risk of bleeding compared to vitamin K antagonists (VKAs). Several studies have shown that DOACs effectively reduce stroke and systemic embolism in AF patients without dementia [16,17,18,19]. However, AF patients with dementia are often underrepresented in these trials, leading to uncertainty about whether these results apply to this group. The 2024 AF guideline from the European Society of Cardiology advises against switching elderly patients stable on warfarin therapy with a well-maintained INR to DOACs in order to maintain clinical stability and reduce the risk of excessive bleeding [1].

Several observational studies have evaluated the use of OAC [5,6,7,8,9,10,11] and compared DOAC to VKA specifically for patients with AF and dementia [20,21,22]. This systematic review and meta-analysis aimed to combine existing data to assess the safety and efficacy of OACs and to determine whether DOACs provide safer and more effective options for AF patients with dementia. The review seeks to address the current knowledge gap and offer better insights into optimal anticoagulant practices for this vulnerable population.

## 2. Materials and Methods

### 2.1. Standard Protocol Approvals and Registrations

This was a systematic review and meta-analysis reported according to the Preferred Reporting Items for Systematic Reviews and Meta-Analyses (PRISMA) statement [23]. The study was limited to published evidence in the English language. Due to the type of study (systematic review), ethical committee approval was not requested. The Population, Intervention, Comparison, and Outcome (PICO) framework was used to develop the research question and identify relevant articles [24]. The study was registered in the International Prospective Register of Systematic Reviews (PROSPERO) with the registration number CRD42024595545.

### 2.2. Data Sources, Searches, and Study Selection

Using the Population, Intervention, Control, Outcome (PICO) format, two reviewers (A.A., A.A.) conducted an independent search within MEDLINE (via PubMed), Web of Science, and Cochrane Library to identify published cohort studies testing DOAC versus VKA in adult patients with atrial fibrillation who are diagnosed with dementia. The search was from inception to 30 October 2024. In addition, the reference lists of included studies were searched. The following search terms were used to identify relevant studies: “Dementia”, “Alzheimer”, “Cognitive Impairment”, “Amentia”, “Vascular Dementia”, “Anticoagulant Drug”, “Anticoagulant Agents”, “Anticoagulation”, “Indirect Thrombin Inhibitors”, “Antithrombins”, “Factor Xa Inhibitors”, “Warfarin”, “NOAC”, “Rivaroxaban”, “Apixaban”, “Edoxaban”, and “Dabigatran”. The complete search strategy can be found in Table S1. Two investigators (A.A. and A.A.) independently assessed the retrieved studies. Disagreements were resolved by consensus or by consulting a third investigator (S.A.).

### 2.3. Quality Control, Bias Assessment, and Data Extraction

The Newcastle–Ottawa Scale was used to assess risk of bias (Table S2) [25]. Quality control and bias assessment were performed by three independent investigators (A.M.A., A.A., and A.A.), and disagreements were resolved by consensus. Data was extracted in structured forms, including the study name, year of publication, population, sample size, patient characteristics, CHA_2_DS_2_-VASc score, HAD-BLED score, follow-up time, OAC type (e.g., apixaban, rivaroxaban, edoxaban, or warfarin), and event type (e.g., mortality, ischemic stroke, major bleeding, or ICH). The minimum considered follow-up period for the occurrence of the outcomes was 6 months.

### 2.4. Outcomes

The primary outcome of interest was the incidence of mortality in patients treated with DOAC versus VKA. The secondary outcomes were the incidence of ischemic stroke, major bleeding, or ICH in patients treated with DOAC versus VKA. An additional analysis to compare OAC versus no OAC for the above-mentioned outcomes was performed. Moreover, a sensitivity analysis to identify any potential significant differences driven by a specific study was performed.

### 2.5. Statistical Analysis

This meta-analysis was performed using Comprehensive Meta-Analysis software (version 3.0) [26]. Study outcomes were considered as dichotomous variables. The association was evaluated using odds ratio (OR) with a 95% confidence interval (95% CI). The values of OR with confidence intervals were derived from the original articles or from a previous meta-analysis where feasible [27]. The results are summarized using forest plots. Heterogeneity was assessed using I^2^ and Cochran Q statistics. The qualitative interpretation of heterogeneity was mild (I^2^ < 25%), moderate (I^2^ = 25% to 50%), and significant (I^2^ > 50%) heterogeneity, respectively. The Q statistic significance level was set at 0.1, and the equivalent z test for each pooled estimate with a two-tailed *p*-value < 0.05 was considered statistically significant.

## 3. Results

### 3.1. Study Selection and Characteristics

**Figure 1:**
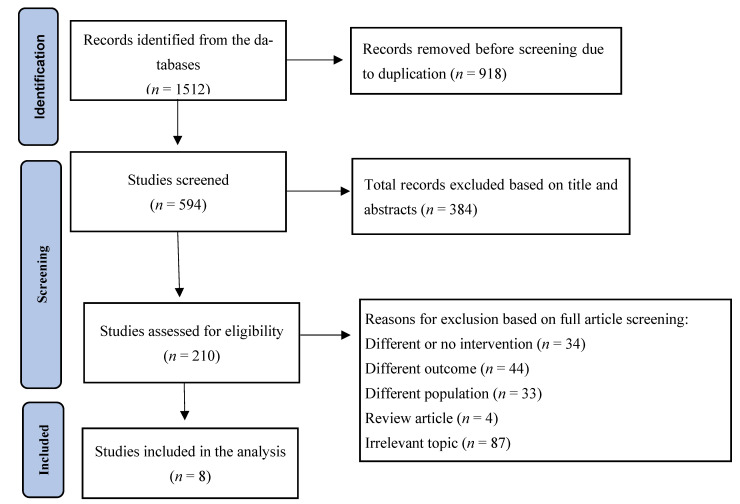
PRISMA flow diagram for selection of studies for the meta-analysis.

A total of 918 duplicate data points were eliminated from the initial search, which yielded a total of 1511 records. After reviewing the titles and abstracts of the remaining 593 entries, we eliminated 384 of them because they were thought to be of inappropriate article types or had no association with atrial fibrillation. We eliminated 34 records because of exposure differences, 33 because of study population differences, 44 because of outcome differences, and 91 because of topic or research type variations after reviewing the full text of the remaining 209 articles. Eight articles were eventually included in the systematic review and meta-analysis after meeting the inclusion criteria. Figure 1 illustrates a graphic representation of the study selection process.

The studies were conducted across different countries, including Spain, the US, the UK, Taiwan, and Sweden, with publication years ranging from 2017 to 2024. The settings varied from healthcare areas and veterans’ systems to nursing homes and dementia registries. The populations included common populations, veterans, nursing home residents, and patients with contraindications to anticoagulation. All the studies were retrospective in design. Sample sizes ranged from 221 to 40,350 participants. The anticoagulant interventions included VKA and DOAC, often compared against oral anticoagulant (OAC) withdrawal. The reported average follow-up periods varied from 1 to 4 years, with significant differences in the proportions of female participants, ranging from 1.4% to 72%. Dementia diagnoses were ascertained using various methods, including RGDS, ICD codes, CPS, and MoCA. Concomitant conditions like diabetes (DM), hypertension (HTN), and heart failure (HF) were prevalent, with rates varying across studies. General clinical characteristics, including CHA_2_DS_2_-VASc and HAD-BLED scores, were reported, providing insights into patient risks (Table 1).

### 3.2. Outcomes

#### 3.2.1. DOAC Versus VKA

##### Effectiveness Outcomes

**Figure 2:**
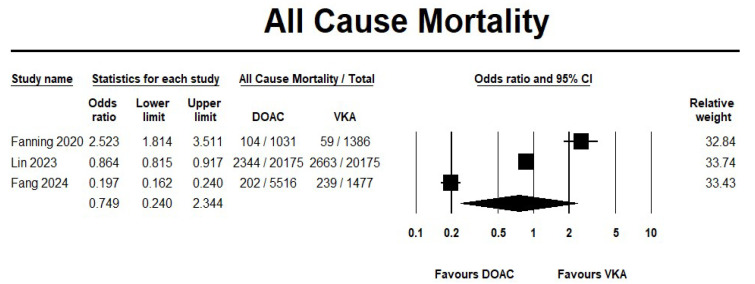
Risk of mortality in direct oral anticoagulant (DOACs) versus vitamin K antagonist (VKA) [20,21,22].

**Figure 3:**
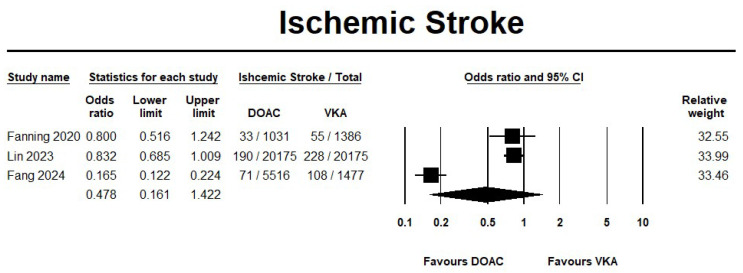
Risk of ischemic stroke in direct oral anticoagulant (DOACs) versus vitamin K antagonist (VKA) [20,21,22].

The use of DOAC showed no significant difference in mortality rates when compared with VKA (OR 0.75, 95% CI 0.24–2.34; I^2^ = 99.2%) (Figure 2). Likewise, the incidence of ischemic stroke did not differ between the two groups (OR 0.47, 95% CI 0.16–1.42; I^2^ = 97.5%) (Figure 3). The sensitivity analyses results were consistent with no differences between the two groups for the mortality and ischemic stroke events. The forest plots for the effectiveness outcomes from the sensitivity analysis are displayed in Figure S1.

##### Safety Outcomes

**Figure 4:**
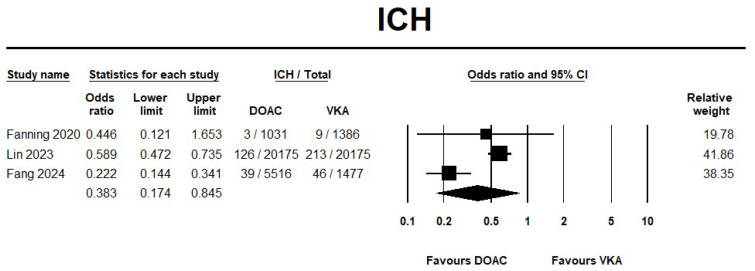
Risk of intracerebral hemorrhage in direct oral anticoagulant (DOAC) versus vitamin K antagonist (VKA) [20,21,22].

**Figure 5:**
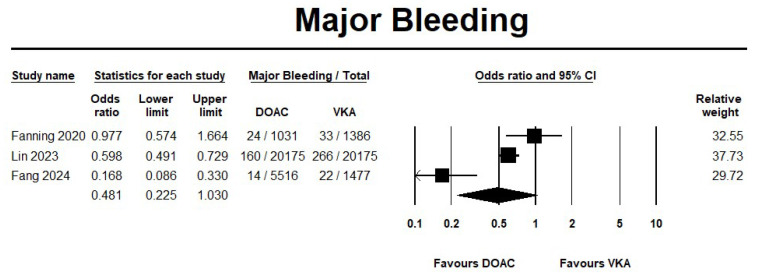
Risk of major bleeding in direct oral anticoagulant (DOAC) versus vitamin K antagonist (VKA) [20,21,22].

The use of DOAC showed a significant reduction by 62% in ICH events compared with VKA (OR 0.38, 95% CI 0.17–0.84; I^2^ = 87.3%) (Figure 4). On the other hand, DOAC showed no significant reduction in major bleeding events when compared with VKA (OR 0.48, 95% CI 0.22–1.03; I^2^ = 88%) (Figure 5). Furthermore, the sensitivity analysis results were consistent with the safety outcomes analysis. The forest plots for the safety outcomes from the sensitivity analysis are displayed in Figure S2.

#### 3.2.2. OAC Versus No OAC

##### Effectiveness Outcomes

**Figure 6:**
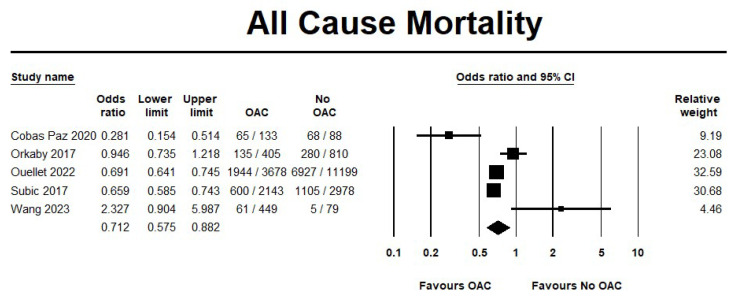
Risk of mortality in oral anticoagulant (OAC) group versus no oral anticoagulant group (no OAC) [11,12,13,14,15].

**Figure 7:**
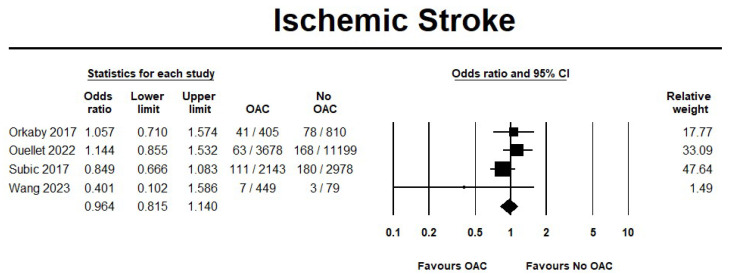
Risk of ischemic stroke in oral anticoagulant (OAC) group versus no oral anticoagulant group (no OAC) [12,13,14,15].

The use of OAC showed a significant reduction in mortality events by 29% when compared to no OAC (OR 0.71, 95% CI 0.57–0.88; I^2^ = 81.3%) (Figure 6). On the other hand, no differences were observed in the ischemic stroke events between the two groups (OR 0.96, 95% CI 0.81–1.14; I^2^ = 27.5%) (Figure 7). Furthermore, the sensitivity analysis results were consistent with the primary analysis (Figure S3).

##### Safety Outcomes

**Figure 8:**
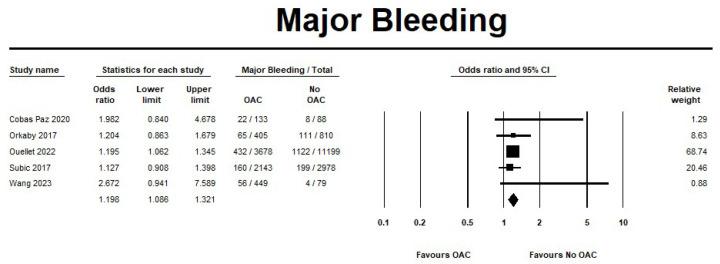
Risk of major bleeding in oral anticoagulant (OAC) group versus no oral anticoagulant group (no OAC) [11,12,13,14,15].

The use of OAC showed an incremental risk in the major bleeding events when compared to no OAC (OR 1.19, 95% CI 1.08–1.3; I^2^ = 0%) (Figure 8). The sensitivity analysis results were consistent with the safety outcomes analysis. The forest plots for the safety outcomes from the sensitivity analysis are displayed in Figure S4.

## 4. Discussion

This systematic review and meta-analysis aimed to assess the efficacy and safety of OAC for the primary prevention of cardioembolic stroke in elderly dementia patients with AF, a population that has been underrepresented in major clinical trials [16,17,18,19] and is not adequately addressed in current guidelines [1,2]. Also, it sought to compare the effectiveness of DOACs with warfarin, focusing on outcomes related to mortality, ischemic stroke, and intracranial hemorrhage risk.

The included studies primarily examined real-world observational data involving dementia atrial fibrillation patients, mostly aged 75 and older, who were either receiving OAC with DOACs or warfarin or had withdrawn from any anticoagulants [11,12,13,14,15,20,21,22]. The primary results indicated favorable mortality outcomes for those who received oral anticoagulants versus those who did not, as well as lower odds of ischemic stroke. OAC initially demonstrated higher odds of major bleeding. Only one study by Cobas et al. addressed the bleeding risk, reporting a HAS-BLED score of 2.9 for their population, indicating a moderate risk of bleeding with only a 4.1% risk of major bleeding [11]. However, sensitivity analysis revealed that excluding this study did not impact the overall results. Our findings partially align with a previous meta-analysis by Wang et al., which indicated that OAC treatment decreases the risk of all-cause mortality in patients with AF and dementia (HR = 0.79, 95% CI: 0.68 to 0.92) [27]. They also reported no significant differences in the risks of major bleeding (HR = 1.12, 95% CI: 0.88–1.42) or ischemic stroke (HR = 0.77, 95% CI: 0.58–1.00).

This meta-analysis updates the previously reported results from Wang et al. by incorporating a recent observational study by Fang et al. [20]. This study compared DOACs with warfarin in AF patients with dementia aged 50 years or older. Fang et al. performed a retrospective review of clinical data for 6993 patients obtained from a national insurance research database covering the years 2009 to 2020 in Taiwan. They utilized 1:1 propensity score matching and Cox proportional hazards models to compare outcomes between warfarin and DOACs. The main strength of this study is its rigorous analysis and larger sample size, evidenced by the narrow confidence interval. Moreover, this study had an extended follow-up period compared to the other two studies [21,22], which monitored participants for less than one year. Fang et al.’s study and the other two included studies demonstrated comparable effect sizes when integrated into the current meta-analysis.

Our findings indicate that there were no differences between DOAC vs. VKA in terms of mortality and ischemic stroke. However, DOACs demonstrated a beneficial outcome by reducing the odds of ICH and major bleeding compared to warfarin. While the reduction in major bleeding did not reach statistical significance, it was deemed significant in previous studies. These findings were already established in landmark trials of DOACs [16,17,18,19] and real-world data among more diverse populations regardless of dementia status [28,29]. However, the clinical significance of our findings showing better safety of DOACs over VKA in patients with dementia deserves emphasis since patients with cognitive impairment are frequently excluded from randomized trials regardless of their risk of thromboembolic or bleeding events, and their anticoagulation management is often challenging due to frailty, adherence, and monitoring limitations.

The results of this meta-analysis suggest that the benefits of oral anticoagulants, particularly DOACs, may be somewhat limited due to existing prescription biases, as indicated by the high heterogeneity in the analysis. However, the reduced adherence requirements and favorable pharmacokinetic properties of DOACs, along with fewer drug and food interactions, may make them more suitable for elderly patients with concurrent dementia, potentially leading to improved clinical outcomes, especially in patients with early to moderate dementia and sufficient social support [30]. Furthermore, some evidence suggests that DOACs may reduce the incidence or the progression of dementia by optimizing cerebral perfusion, minimizing microembolic events, which could lead to a reduction in clinical and subclinical stroke [31,32,33]. However, concerns remain that the use of anticoagulants, including DOACs, in patients with cerebral amyloid angiopathy could lead to cerebral microbleeds and a decline in cognitive status [34]. This highlights the importance of careful risk-benefit evaluation and personalized anticoagulation strategies in this high-risk population. In addition to assessing stroke and bleeding risk scores (CHA_2_DS_2_-VASc and HAS-BLED), clinicians should consider dementia severity, functional status, life expectancy, and social support. Treatment decisions regarding the anticoagulation strategy should be regularly assessed, especially in the setting of cognitive decline progression or a change in the goal of care.

This meta-analysis has several limitations. First, all the studies included were retrospective cohort studies, inherently susceptible to baseline imbalances. While adjustments for multiple variables were made, these adjustments do not eliminate biases introduced by confounding factors. Currently, no RCTs are available due to difficulties ensuring participant adherence and ethical considerations. Additionally, none of the studies addressed the adherence of the populations involved. It is unclear whether individuals in the warfarin group consistently maintained their INR within the therapeutic range or if those in the DOACs group received appropriate dose adjustments based on renal function, weight, and age. The absence of further analyses stratified by DOAC dosing (standard versus reduced) represents another limitation of the study, which is very important to consider in elderly patients with AF. Furthermore, there was considerable heterogeneity among the studies, with variations in study populations and proportions of DOAC medications received. Removing these highly heterogeneous studies could affect the overall findings. Finally, there was a wide variation in the follow-up periods among the included studies, which may have influenced the main outcomes, such as stroke rates and mortality. Additional research is needed to clarify the risk-benefit ratio of anticoagulation for patients with dementia and to confirm the benefits of DOACs compared to warfarin.

## 5. Conclusions

Our findings indicate that DOACs offer a safer profile regarding ICH in dementia patients with AF compared to VKAs, with no significant differences in mortality, ischemic stroke, or major bleeding rates. When comparing the use of OAC to no OAC, the use of OAC showed a significant reduction in mortality events compared to no OAC, with an increased rate of major bleeding. This study highlights the need for careful anticoagulant selection in this vulnerable population.

## Figures and Tables

**Table 1:** Summary of studies that were included in the systematic review and meta-analysis.

Study	Country	Year	Population	Design	Sample Size	Intervention	Comparator	Follow-Up (Years)	Female (%)	Dementia Diagnosis	Ischemic Stroke (%)	DM (%)	HTN (%)	HF (%)
Cobas Paz 2020 [11]	Spain	2020	Common population	Retrospective	221	VKA/DOAC	OAC withdrawal	2.8	69.7	RGDS (5–7)	18.1	18.1	65.6	18.1
Orkaby 2017 [12]	US	2017	Veterans	Retrospective	1215	VKA	OAC withdrawal	4	1.4	ICD-9 code	25.4	45.2	95	59.2
Ouellet 2022 [13]	US	2022	Nursing home residents	Retrospective	14,877	VKA/DOAC	OAC withdrawal	1	72	CPS (5–6)	55.2	38	89.7	42.5
Subic 2017 [14]	Sweden	2017	Common population	Retrospective	5121	VKA	OAC withdrawal	1.7	47.2	ICD-10 code	22.7	19.4	62.8	37.2
Wang 2023 [15]	US	2023	Patients with contraindications to anticoagulation were excluded	Retrospective	528	VKA/DOAC	OAC withdrawal	2	49.9	MoCA (<23)	9.8	27.8	90.9	37.2
Fanning 2020 [21]	UK	2020	Common population	Retrospective	2399	DOAC	VKA	0.8	54	Not mentioned	27.3	22.2	68.3	13.6
Lin 2023 [22]	US	2023	Common population	Retrospective	40,350	DOAC	VKA	0.5	59.5	ICD-9/10 code	42.5	42.9	92.2	83.5
Fang 2024 [20]	Taiwan	2024	Common population	Retrospective	6993	DOAC	VKA	NR	56.8	ICD-9/10 code	1.95	12.4	31.7	74.3

Abbreviations: VKA: “vitamin K antagonist”, DOAC: “direct oral anticoagulant”, OAC: “oral anticoagulation”, RGDS: “Reisberg Global Deterioration Scale”, ICD: “International Classification of Diseases”, CPS: “Cognitive Performance Scale”, MoCA: “Montreal Cognitive Assessment”, DM: “Diabetes Mellitus”, HTN: “hypertension”, HF: “heart failure”, NR: “Not Reported”.

## Data Availability

All data generated or analyzed during this study are included in this article and its Supplementary Materials files.

## Supplementary Materials

The following supporting information can be downloaded at https://www.mdpi.com/article/10.3390/jcm14165758/s1. Table S1: Keywords and search results; Table S2: Newcastle–Ottawa scale for assessing the quality of included studies; Figure S1: Effectiveness outcomes from the sensitivity analysis (DOAC versus VKA); Figure S2: Safety outcomes from the sensitivity analysis (DOAC versus VKA); Figure S3: Effectiveness outcomes from the sensitivity analysis (OAC vs no OAC); Figure S4: Safety outcomes from the sensitivity analysis (OAC vs no OAC).

## References

[1] Van Gelder I.C., Rienstra M., Bunting K.V., Casado-Arroyo R., Caso V., Crijns H.J.G.M., De Potter T.J.R., Dwight J., Guasti L., Hanke T. (2024). 2024 ESC Guidelines for the management of atrial fibrillation developed in collaboration with the European Association for Cardio-Thoracic Surgery (EACTS). Eur. Heart J..

[2] Joglar J.A., Chung M.K., Armbruster A.L., Benjamin E.J., Chyou J.Y., Cronin E.M., Deswal A., Eckhardt L.L., Goldberger Z.D., Gopinathannair R. (2024). 2023 ACC/AHA/ACCP/HRS Guideline for the Diagnosis and Management of Atrial Fibrillation: A Report of the American College of Cardiology/American Heart Association Joint Committee on Clinical Practice Guidelines. Circulation.

[3] Khurshid S., Ashburner J.M., Ellinor P.T., McManus D.D., Atlas S.J., Singer D.E., Lubitz S.A. (2023). Prevalence and Incidence of Atrial Fibrillation Among Older Primary Care Patients. JAMA Netw. Open.

[4] Rosas Diaz A.N., Troy A.L., Kaplinskiy V., Pritchard A., Vani R., Ko D., Orkaby A.R. (2024). Assessment and Management of Atrial Fibrillation in Older Adults with Frailty. Geriatrics.

[5] Koh Y.H., Lew L.Z.W., Franke K.B., Elliott A.D., Lau D.H., Thiyagarajah A., Linz D., Arstall M., Tully P.J., Baune B.T. (2022). Predictive role of atrial fibrillation in cognitive decline: A systematic review and meta-analysis of 2.8 million individuals. Europace.

[6] Papanastasiou C.A., Theochari C.A., Zareifopoulos N., Arfaras-Melainis A., Giannakoulas G., Karamitsos T.D., Palaiodimos L., Ntaios G., Avgerinos K.I., Kapogiannis D. (2021). Atrial Fibrillation Is Associated with Cognitive Impairment, All-Cause Dementia, Vascular Dementia, and Alzheimer’s Disease: A Systematic Review and Meta-Analysis. J. Gen. Intern. Med..

[7] Giannone M.E., Filippini T., Whelton P.K., Chiari A., Vitolo M., Boriani G., Vinceti M. (2022). Atrial Fibrillation and the Risk of Early-Onset Dementia: A Systematic Review and Meta-Analysis. J. Am. Heart Assoc..

[8] Dodson J.A., Petrone A., Gagnon D.R., Tinetti M.E., Krumholz H.M., Gaziano J.M. (2016). Incidence and Determinants of Traumatic Intracranial Bleeding Among Older Veterans Receiving Warfarin for Atrial Fibrillation. JAMA Cardiol..

[9] Jankowska-Polańska B., Katarzyna L., Lidia A., Joanna J., Dudek K., Izabella U. (2016). Cognitive function and adherence to anticoagulation treatment in patients with atrial fibrillation. J. Geriatr. Cardiol..

[10] Nishimura S., Kumamaru H., Shoji S., Nakatani E., Yamamoto H., Ichihara N., Sandhu A.T., Miyachi Y., Miyata H., Kohsaka S. (2023). Frailty and subsequent adverse outcomes in older patients with atrial fibrillation treated with oral anticoagulants: The Shizuoka study. Res. Pract. Thromb. Haemost..

[11] Cobas Paz R., Raposeiras Roubín S., Abu Assi E., Pardal C.B., Comesaña J.G., López A.G.-C., Queija B.C., Fernández M.C., Pousa I.M., Erquicia P.D. (2020). Impact of anticoagulation in patients with dementia and atrial fibrillation. Results of the CardioCHUVI-FA registry. Rev. Esp. Cardiol. (Engl. Ed.).

[12] Orkaby A.R., Ozonoff A., Reisman J.I., Miller D.R., Zhao S., Rose A.J. (2017). Continued Use of Warfarin in Veterans with Atrial Fibrillation After Dementia Diagnosis. J. Am. Geriatr. Soc..

[13] Ouellet G.M., O’Leary J.R., Leggett C.G., Skinner J., Tinetti M.E., Cohen A.B. (2023). Benefits and harms of oral anticoagulants for atrial fibrillation in nursing home residents with advanced dementia. J. Am. Geriatr. Soc..

[14] Subic A., Cermakova P., Religa D., Han S., von Euler M., Kåreholt I., Johnell K., Fastbom J., Bognandi L., Winblad B. (2018). Treatment of Atrial Fibrillation in Patients with Dementia: A Cohort Study from the Swedish Dementia Registry. J. Alzheimers. Dis..

[15] Wang W., Lessard D., Kiefe C.I., Goldberg R.J., Parish D., Helm R., Trymbulak K., Mehawej J., Abu H., Bamgbade B.A. (2023). Differential effect of anticoagulation according to cognitive function and frailty in older patients with atrial fibrillation. J. Am. Geriatr. Soc..

[16] Granger C.B., Alexander J.H., McMurray J.J., Lopes R.D., Hylek E.M., Hanna M., Al-Khalidi H.R., Ansell J., Atar D., Ave-zum A. (2011). Apixaban versus warfarin in patients with atrial fibrillation. N. Engl. J. Med..

[17] Giugliano R.P., Ruff C.T., Braunwald E., Murphy S.A., Wiviott S.D., Halperin J.L., Waldo A.L., Ezekowitz M.D., Weitz J.I., Špinar J. (2013). Edoxaban versus warfarin in patients with atrial fibrillation. N Engl J Med..

[18] Patel M.R., Mahaffey K.W., Garg J., Pan G., Singer D.E., Hacke W., Breithardt G., Halperin J.L., Hankey G.J., Piccini J.P. (2011). Rivaroxaban versus warfarin in nonvalvular atrial fibrillation. N Engl J Med..

[19] Connolly S.J., Ezekowitz M.D., Yusuf S., Eikelboom J., Oldgren J., Parekh A., Pogue J., Reilly P.A., Themeles E., Varrone J. (2009). Dabigatran versus warfarin in patients with atrial fibrillation. N. Engl. J. Med..

[20] Fang C.-W., Hsieh C.-Y., Yang H.-Y., Tsai C.-F., Sung S.-F. (2024). Comparative effectiveness and safety of direct oral anticoagulants and warfarin in atrial fibrillation patients with dementia. Eur. Stroke J..

[21] Fanning L., Lau W.C.Y., Mongkhon P., Man K.K.C., Bell J.S., Ilomäki J., Dārziņš P., Lau K.K., Wei L., Wong L.C.K. (2020). Safety and effectiveness of direct oral anticoagulants vs. warfarin in people with atrial fibrillation and dementia. J. Am. Med. Dir. Assoc..

[22] Lin K.J., Singer D.E., Bykov K., Bessette L.G., Mastrorilli J.M., Cervone A., Kim D.H. (2023). Comparative effectiveness and safety of oral anticoagulants by dementia status in older patients with atrial fibrillation. JAMA Netw..

[23] Page M.J., McKenzie J.E., Bossuyt P.M., Boutron I., Hoffmann T.C., Mulrow C.D., Shamseer L., Tetzlaff J.M., Moher D. (2021). Updating guidance for reporting systematic reviews: Development of the PRISMA 2020 statement. J. Clin. Epidemiol..

[24] Schardt C., Adams M.B., Owens T., Keitz S., Fontelo P. (2007). Utilization of the PICO framework to improve searching PubMed for clinical questions. BMC Med. Inform. Decis. Mak..

[25] Wells G., Shea B., O’Connell J. (2014). The Newcastle-Ottawa Scale (NOS) for Assessing the Quality of Nonrandomised Studies in Meta-analyses. Ott. Hosp. Res. Inst..

[26] Bruggemann P., Rajguru K. (2022). Comprehensive meta-analysis (CMA) 3.0: A software review. J. Market. Anal..

[27] Wang D., Xu X., Han X., Xie J., Zhou H., Peng W., Pan G. (2023). Clinical benefits of oral anticoagulants in atrial fibrillation patients with dementia: A systematic review and meta-analysis. Front. Cardiovasc. Med..

[28] Yang L., Brooks M.M., Glynn N.W., Zhang Y., Saba S., Hernandez I. (2020). Real-World Direct Comparison of the Effectiveness and Safety of Apixaban, Dabigatran, Rivaroxaban, and Warfarin in Medicare Beneficiaries with Atrial Fibrillation. Am. J. Cardiol..

[29] Xian Y., Xu H., O’Brien E.C., Shah S., Thomas L., Pencina M.J., Fonarow G.C., Olson D.M., Schwamm L.H., Bhatt D.L. (2019). Clinical Effectiveness of Direct Oral Anticoagulants vs Warfarin in Older Patients With Atrial Fibrillation and Ischemic Stroke: Findings From the Patient-Centered Research Into Outcomes Stroke Patients Prefer and Effectiveness Research (PROSPER) Study. JAMA Neurol..

[30] Ruiz Vargas E., Sposato L.A., Lee S.A.W., Hachinski V., Cipriano L.E. (2018). Anticoagulation Therapy for Atrial Fibrillation in Patients With Alzheimer’s Disease. Stroke.

[31] Pendlebury S.T. (2021). Direct Oral Anticoagulants and Prevention of Dementia in Nonvalvular Atrial Fibrillation. Stroke.

[32] Latif F., Nasir M.M., Meer K.K., Farhan S.H., Cheema H.A., Khan A.B., Umer M., Rehman W.U., Ahmad A., Khan M.A. (2024). The effect of oral anticoagulants on the incidence of dementia in patients with atrial fibrillation: A systematic review and meta-analysis. Int. J. Cardiol. Cardiovasc. Risk Prev..

[33] Zhang C., Zhang J., Zhao X., Jiang D., Liu X., Liang Y. (2024). Association of direct oral anticoagulants and warfarin with incidence of dementia in atrial fibrillation patients: A systematic review and meta-analysis. Int. J. Cardiol. Heart Vasc..

[34] Fusco L., Palamà Z., Scarà A., Borrelli A., Robles A.G., Luca G.D.M.D., Romano S., Sciarra L. (2024). Management of cerebral amyloid angiopathy and atrial fibrillation: We are still far from precision medicine. World J. Cardiol..

