# Supplementary material for: Comparison of Safety and Effectiveness Between Direct Oral Anticoagulants and Vitamin K Antagonists in Dementia Patients with Atrial Fibrillation: A Systematic Review and Meta-Analysis
Source: J Clin Med. 2025 Aug 14;14(16):5758. doi: 10.3390/jcm14165758 (PMC12386684; doi:10.3390/jcm14165758)
Supplement: Supplementary file 1 [file jcm-14-05758-s001.zip › jcm-3791651-supplementary.pdf]

Table S1: Keywords and search results

Database	Keyword	Filter	Date	Results
PubMed	((Dementia[Title/Abstract]) OR (Alzheimer[Title/Abstract]) OR (Cognitive impairment[Title/Abstract]) OR (Amentia[Title/Abstract]) OR (Amentia[Title/Abstract]) OR (Vascular Dementia[Title/Abstract])) AND ((Anticoagulant Drug[Title/Abstract]) OR (Anticoagulant Agents[Title/Abstract]) OR (Anticoagulation[Title/Abstract]) OR (Indirect Thrombin Inhibitors[Title/Abstract]) OR (Antithrombins[Title/Abstract]) OR (Factor Xa Inhibitors[Title/Abstract]) OR (Warfarin[Title/Abstract]) OR (NOAC[Title/Abstract]) OR (Rivaroxaban[Title/Abstract]) OR (Apixaban[Title/Abstract]) OR (Edoxaban[Title/Abstract]) OR (Dabigatran[Title/Abstract]))	NA	October 30th, 2024	591
Web of Science	#1 TS= (Dementia OR Alzheimer OR Cognitive impairment OR Amentia OR Vascular Dementia OR Lewy Body Disease)<br><br>#2 TS= (Anticoagulant Drug OR Anticoagulant Agents OR Anticoagulation	NA	October 30th, 2024	919

	OR Indirect Thrombin Inhibitors OR Antithrombins OR Factor Xa Inhibitors OR Warfarin OR NOAC OR Rivaroxaban OR Apixaban OR Edoxaban OR Dabigatran)<br><br>#3 #1 AND #2			
Cochrane Library	((Dementia) OR (Cognitive impairment) OR (Amentia) OR (Vascular Dementia) OR (Lewy Body Disease) OR (Alzheimer)) AND ((Anticoagulant Drug) OR (Anticoagulant Agents) OR (Anticoagulation) OR (Indirect Thrombin Inhibitors) OR (Antithrombins) OR (Warfarin) OR (NOAC) OR (Rivaroxaban) OR (Apixaban) OR (Edoxaban) OR (Dabigatran))	Title<br><br>Abstract<br><br>keyword	October 30th, 2024	1

Table S2: Newcastle–Ottawa scale for assessing the quality of included studies

**Study**	**Selection**	**Comparability**	**Outcome**	**Score**

	Representativeness of the exposed cohort	Selection of the non-exposed cohort	Ascertainment of exposure	Demonstration that outcome of interest was not present at start of study	Comparability of cohorts on the basis of the design or analysis	Assessment of outcome	Was follow-up long enough for outcomes to occur	Adequacy of follow up of cohorts	
**Orkaby, 2017**	-	✓	-	✓	✓	✓	✓	-	**5**
**Subic, 2017**	✓	✓	-	✓	-	✓	✓	-	**5**
**Fanning, 2020**	✓	✓	-	✓	-	✓	✓	-	**5**
**Cobas Paz, 2020**	✓	✓	-	✓	✓✓	-	✓	-	**6**
**Ouellet, 2022**	-	✓	-	✓	✓	✓	✓	-	**5**
**Lin, 2023**	✓	✓	-	✓	-	✓	✓	-	**5**
**Wang, 2023**	-	✓	-	✓	-	✓	✓	-	**4**
**Fang, 2024**	✓	✓	✓	✓	✓✓	✓	✓	✓	**9**

All Cause Mortality

Study name	Point	Lower limit	Upper limit
Fanning 2020	0.414	0.097	1.764
Lin 2023	0.702	0.058	8.553
Fang 2024	1.458	0.511	4.165
	0.749	0.240	2.344

Odds ratio (95% CI) with study removed

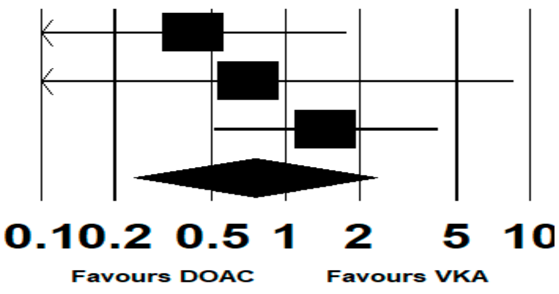

Ischemic Stroke

Study name	Point	Lower limit	Upper limit
Fanning 2020	0.372	0.076	1.815
Lin 2023	0.361	0.077	1.692
Fang 2024	0.827	0.692	0.987
	0.478	0.161	1.422

Odds ratio (95% CI) with study removed

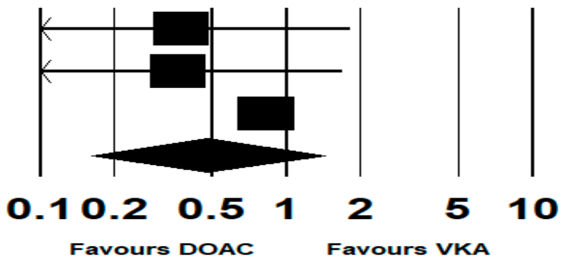

Figure S1: Effectiveness outcomes from the sensitivity analysis (**DOAC versus VKA) [20–22]**

ICH

Study name	Point	Lower limit	Upper limit
Fanning 2020	0.368	0.141	0.958
Lin 2023	0.237	0.158	0.357
Fang 2024	0.584	0.470	0.727
	0.383	0.174	0.845

Odds ratio (95% CI) with study removed

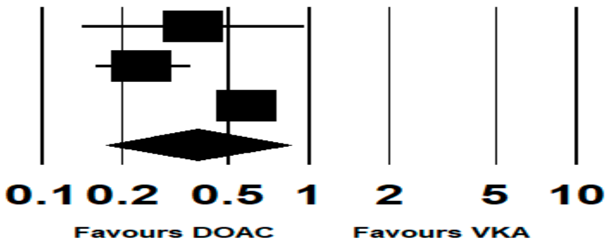

Major Bleeding

Study name	Point	Lower limit	Upper limit
Fanning 2020	0.331	0.096	1.144
Lin 2023	0.411	0.073	2.301
Fang 2024	0.717	0.451	1.139
	0.481	0.225	1.030

Odds ratio (95% CI) with study removed

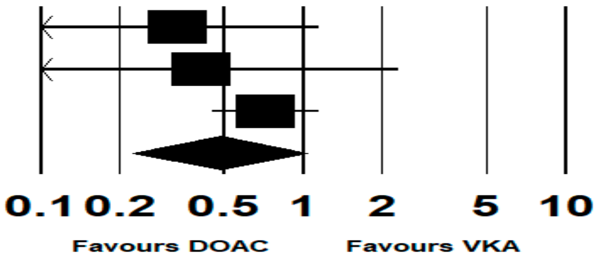

Figure S2: Safety outcomes from the sensitivity analysis (**DOAC versus VKA) [20–22]**

All Cause Mortality

Study name

Study name	Point	Lower limit	Upper limit
Cobas Paz 2020	0.761	0.635	0.911
Orkaby 2017	0.655	0.521	0.823
Ouellet 2022	0.731	0.467	1.143
Subic 2017	0.740	0.488	1.124
Wang 2023	0.681	0.563	0.824
	0.712	0.575	0.882

Odds ratio (95% CI) with study removed

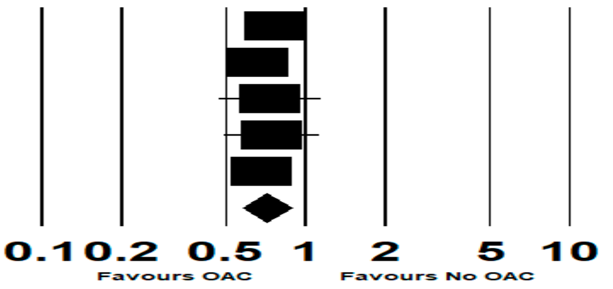

Ischemic Stroke

Study name

Study name	Point	Lower limit	Upper limit
Orkaby 2017	0.944	0.785	1.137
Ouellet 2022	0.885	0.721	1.087
Subic 2017	1.081	0.857	1.364
Wang 2023	0.976	0.824	1.157
	0.964	0.815	1.140

Odds ratio (95% CI) with study removed

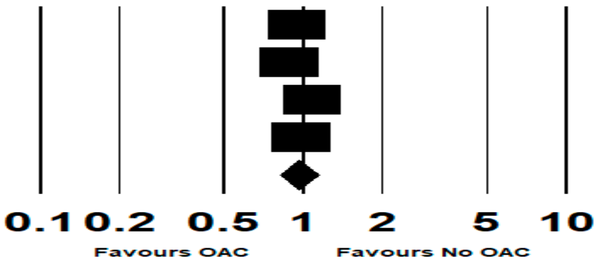

Figure S3: Effectiveness outcomes from the sensitivity analysis (OAC vs no OAC) [11–15]

Major Bleeding

Study name	Point	Lower limit	Upper limit
Cobas Paz 2020	1.190	1.079	1.313
Orkaby 2017	1.197	1.081	1.326
Ouellet 2022	1.203	1.011	1.433
Subic 2017	1.217	1.091	1.358
Wang 2023	1.189	1.078	1.312
	1.198	1.086	1.321

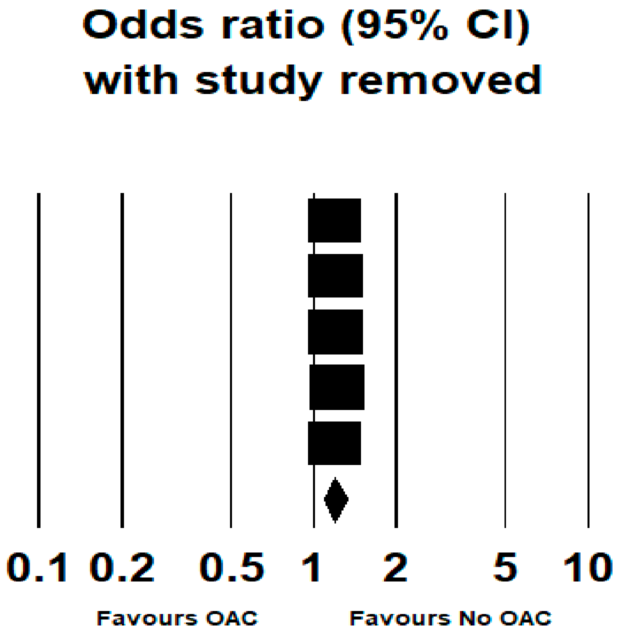

Figure S4: Safety outcomes from the sensitivity analysis (**OAC vs no OAC**) [11–15]
